# Transforming narratives of physician identity formation and healing: a longitudinal qualitative study of physicians’ stories about spirituality and medicine, from residency to practice

**DOI:** 10.1186/s12909-025-06788-6

**Published:** 2025-02-27

**Authors:** Gowri Anandarajah, Georgia Sleeth, Meera Mennillo, Achutha Srinivasan

**Affiliations:** 1 Warren Alpert Medical School of Brown University, 222 Richmond Street, Providence, RI USA; 2 Internal Medicine Residency, University of Washington, Seattle, WA USA; 3 University of Rhode Island, Kingston, RI USA

**Keywords:** Physician identity formation, Burnout, Resilience, Spiritual health, Wellbeing, Narrative medicine

## Abstract

**Background:**

Concern about burnout has prompted increased attention on fostering physician resilience throughout the educational continuum. Studies indicate that lack of meaning-making and connection (domains of spiritual wellbeing) place physicians at risk for burnout. While evidence support including spiritual care in comprehensive patient care to help patients/families heal from impactful experiences, few studies explore physicians’ spiritual wellbeing as they routinely confront suffering and death in their daily work. Storytelling taps into spiritual aspects of human experience. This study, unique in the literature, examined the stories physicians chose to tell about spirituality and medicine over 20-years, from trainee to practicing physician, to explore how these experiences impact professional development and wellbeing.

**Methods:**

*Design:* Qualitative individual interview study – secondary analysis of a rich dataset of physician interviews, gathered over 20-years beginning in first-year residency, regarding attitudes and approach to spiritual care. For this new study, researchers extracted and analyzed the previously unexplored stories participants spontaneously told during interviews.

*Setting*: Participants completed the same USA residency program and now practice throughout USA and Canada.

*Participants:* In study-year 1, all residents (PGY1,2,3) participated; response rate (RR) 97%, reflected a diversity of personal beliefs (atheist to religious). Researchers followed the PGY1 class for 20-years (2001–2020), interviewing them in study-years 1, 3, 11 and 20 (RR 100%, 100%, 97%, 54%).

*Data Collection*: Researchers extracted stories from interview transcripts.

*Analysis:* 4 researchers analyzed 204 stories from 66 interviews with 34 physicians, using grounded theory.

**Results:**

Irrespective of personal spiritual beliefs, trainees and practicing physicians told numerous spirituality-related stories. Longitudinal story themes—(1) Dissonance to Integration, (2) Formation and Transformation, and (3) Accidental to Purposeful Healing – reflected physicians’ ongoing spiritual journeys as they grappled with meaning, values, purpose, and connection in their daily work. Spiritually impactful moments, whether distressing or uplifting, occurred throughout physicians’ careers influencing professional/personal development, resilience and clinical approach. Spiritual practices (religious/secular) and reflection fostered healing for patients/families and physicians.

**Conclusion:**

Physicians’ longitudinal spiritual-care stories provide new insights into their professional/personal development. Reflection on spiritually impactful moments, both distressing and uplifting, may trigger transformative learning towards meaning-making, resilience, burnout prevention and positive physician identity formation.

## Background

Concern regarding physician burnout, intensified by the COVID-19 pandemic, has spurred increasing interest in physician wellbeing [1–9], including how to foster the development of wellbeing and resilience in trainees, as part of their professional identity formation [10–18]. Recent studies indicate that an inability to find meaning in work or feelings of isolation place physicians at risk for burnout [19–21]. Meaning-making and feelings of connection are two vital aspects of spiritual wellbeing [22, 23]. However, the role of spiritual health in physicians’ overall wellbeing and professional formation has been largely neglected in the medical literature.

Defining spirituality is challenging because of its multidimensional nature [22, 23] and the diverse ways people express spirituality. Consensus in the medical literature is to take a broad, inclusive view of spiritual health as a universal human need, like physical and mental health [22, 23]. Commonly discussed domains include: cognitive/existential aspects (eg. meaning, purpose, values, beliefs); experiential aspects (eg. connection to self/others/community/nature/the transcendent, compassion for self/others, inner strength/resilience/peace); and behavioral aspects (eg. life choices, practices to improve spiritual wellbeing, such as prayer, meditation or walks in nature) [22–25].

Spiritual distress, a term used in clinical chaplaincy and medical contexts, occurs “when individuals are unable to find sources of meaning, hope, love, peace, comfort, strength and connection in life or when conflict occurs between their beliefs and what is happening in their life.” [26] Similarly, studies from the psychoanalytical literature indicate that spiritual struggles are common following stressful events [27, 28] and are: “among the deepest of all conflicts… [that] can shake us to the heart of our being.” [27] Pargament defines spiritual struggles as “experiences of tension, conflict, or strain that center on whatever people view as sacred,” [27] explaining that “sacred” can have secular and/or religious meaning for different individuals. For example, “for some, a job is simply a way to put food on the table … [for others] work is less a job than it is a calling or a vocation, a source of ultimate purpose in life.” [27] Addressing spiritual struggles can prevent progression of usual stress to post-traumatic stress, depression and/or anxiety [27, 29].

In this context, two of the three domains in Maslach’s Burnout Inventory [19] – depersonalization and lack of self-efficacy – relate to spiritual struggles. The self-efficacy domain, applied to physicians, includes both the belief that they have the requisite skills for the job and that their work/calling has meaning and purpose [20, 21, 27, 30, 31].

In clinical practice, growing evidence supporting the role of spiritual care in comprehensive patient care [32–39] has prompted calls for expanding the biopsychosocial model to a biopsychosocial-spiritual model of care [22, 40, 41]. Currently, guidelines for hospital accreditation, medical education, and clinical organizations worldwide include consideration of spiritual health, including addressing spiritual distress [24, 25, 42–50]. Many studies explore spiritual needs and beliefs of patients [51–55]. However, most studies of physicians focus on barriers to addressing patients’ spiritual needs, rather than the spiritual wellbeing of physicians themselves, as they routinely confront suffering and death [56–65].

Unlike other areas of health, where direct questioning regarding symptoms leads to diagnoses, spiritual concerns are often expressed in the form of metaphor or story [66–69]. Thus, understanding how spiritual factors influence an individual’s health or healthcare decisions can be enhanced by lessons learned from the field of narrative medicine, which explores the stories of patients, caregivers, and healthcare providers [51, 70–74].

While several studies and curricula focus on resilience, well-being and compassion in trainees [10–18], less is known about how practicing physicians, the mentors for our trainees, make meaning and grow as their careers progress [75, 76]. Similarly, while much is written about physicians’ narratives [70, 77], few, if any, studies explicitly explore the spiritual dimension of these narratives.

Over 20-years we compiled a dataset of in-depth, individual interviews with physicians about their attitudes and approaches to spirituality and patient care. One sub-group was interviewed four times from first-year residency to 20-years later. Previous studies from this dataset include one at 10-years, examining effects of a residency spiritual care curriculum [78], and one at 20-years (parent study), analyzing factors influencing physicians’ ability to provide spiritual care [79]. This rich dataset included numerous impactful physician stories that had not been explored.

The purpose of this study was to perform a new qualitative analysis of this dataset focused *solely* on the stories physicians chose to tell during their interviews. We hypothesized that examining stories alone, rather than cognitive level answers to questions, could reveal deeper insights into physicians’ experiences regarding spirituality and patient care, including meaning making, connection, and resilience, as participants developed from residents to mid-career physicians. To our knowledge no other study has examined physicians’ narratives in this way. Therefore, our study question was exploratory in nature: What do physicians’ spontaneous stories about spirituality and patient care over 20-years tell us about their experiences and development as physicians?

## Methods

### Design

This longitudinal, individual interview, qualitative study was a secondary analysis of interviews collected for a parent study over 20-years [79]. For this new study, we extracted and analyzed the stories physicians spontaneously told, as they answered questions. We selected a grounded theory approach to gain insights into physicians’ inner experiences, learning and development through their stories.

### Setting

All participants attended the same family medicine residency in Northeastern USA. Over 20-years participants have practiced throughout North America and internationally.

### Participants

The parent study’s goal [79] was to capture a broad range of opinions, including outliers. Therefore, all 38 resident physicians (PGY1, 2 and 3) enrolled during academic-year 2000–2001 (study-year 1) were invited to participate – corresponding to Batch 1, 2, and 3 in this current study. None of these study-year 1 residents had received spiritual care training during residency. A subset of this full group of participants, the PGY1 class (Class of 2003; *N* = 13), was interviewed longitudinally: as PGY1s in study-year 1 (2000–2001; Batch 1), PGY3s in study-year 3 (2003; Batch 4), and practicing physicians in study-years 11 (2011; Batch 5) and 20 (2020; Batch 6). After study-year 1, the longitudinal study-group received spiritual care training during residency.

### Procedures

Individual, 30–60 min, semi-structured interviews, using an interview guide, were audio-recorded and transcribed verbatim with identifiers removed. Questions explored: beliefs regarding spirituality and medicine, role of physicians, and clinical approach to spiritual care (details previously published) [79]. To maximize inclusivity, we did not provide definitions of spirituality or religion, but rather asked participants to provide their own definitions. For this new study, two researchers (AS, GS) extracted all stories from interviews and organized them into 6 batches by study-year and training-year. A story was defined as a description of a *specific* event, person, or experience.

### Analysis

We assembled a new 4-member research team (only one member from parent study), to minimize bias in analysis. Our multi-step inductive analysis approach was based on grounded theory [80, 81]. First, all 4 researchers (GA, GS, MM, AS) independently analyzed batch-1 stories and met for a group analysis meeting to reach consensus on the initial code list. We then conducted iterative rounds of analysis of each of the 6 batches of stories. For each batch, this involved independent analysis followed by group analysis meetings for rigorous discussion and exchange of data interpretation. The code list was modified as needed to accommodate evolution in the stories (open coding).

Next, we conducted group analysis meetings to reach consensus on themes, subthemes, and evolution over time. Evolution over time was determined by comparing each of the 4 batches of stories from the longitudinal study group as well as examining each physician’s stories over time. Then, we held, multiple analysis meetings to determine relationships between themes (axial and selective coding), which culminated in a theoretical model illustrating these relationships. Finally, all researchers contributed representative quotations, ensuring maximum participant representation. Throughout this process, researchers disclosed and clarified biases that might impact analysis.

After we had completed the above in-depth grounded theory thematic analysis, we conducted simple descriptive analyses of participants’ characteristics and each story’s characteristics (topics and characters) in order to provide context for the findings. These elements were compiled after the grounded theory analysis to minimize bias in story interpretation.

### Ethical review

We obtained Institutional Review Board (IRB) approval for every step of this qualitative longitudinal study. Please see Ethical Approval and Consent to Participate statement.

## Results

### General

We analyzed 204 stories from 66 interviews with 34 physicians. In study-year 1 the response rate (RR) was 97% (37/38); 3 interviews were lost (poor recordings), therefore 34 were included (89%). For the physicians followed longitudinally, RRs were 100% in study-year 1, 100% in study-year 3, 97% in study-year 11, and 54% in study-year 20.

### Participant characteristics

We collected limited demographics (Table 1) to protect participant anonymity, given their status as trainees in study-year 1 and the potentially sensitive topic of spirituality. The longitudinal study-group and full sample were similar in age as PGY3s, gender (77% F), and personal beliefs. Spiritual/religious beliefs were diverse, ranging from atheist to very religious. Approximately 30% of both groups described themselves as “spiritual, not religious” and 15% as “not spiritual or religious.” Almost all participants told stories.

**Table 1:** Participant characteristics

		**Longitudinal Study Group** ***N*=13**		**Full Group** ***N*=34**
**Personal importance of spirituality or religion**	Not Spiritual or Religious	2 (15%)		5 (15%)
	Spiritual, Not Religious	4 (30%)		10 (29%)
	Religious	5 (38%)		15 (44%)
	Searching	2 (15%)		3 (9%)
	Unknown	-		1 (3%)
**Religious or Belief Background**	Christian	5 (38%)		19 (56%)
	Jewish	4 (31%)		5 (15%)
	Buddhist	-		1 (3%)
	No religion identified	4 (31%)		7 (21%)
	Atheist	-		1 (3%)
**Years in Practice**		1-20		1-3
**Current and Previous Practice Locations**^**a**^	USA States	Arizona California Massachusetts New York Rhode Island		Rhode Island
	International	Canada Nepal East Africa		
**Current and Previous Practice Types**^a^		Private Practice Hospital-based practice Hospice & Palliative Care Indian Health Service Planned Parenthood Student Health Services	Community Health Centers Academic/Teaching Urgent Care Department of Corrections Global Health Settings State Health Department Positions	All – Residency (heavily hospital-based with strong outpatient continuity patient panel)

All data extracted from volunteered interview responses

^a^By 20 years, many physicians had had several different practice experiences

### Story characteristics

Stories reflected participants’ varied definitions of spirituality and were commonly about end-of-life (*N* = 64; 31%), patients’ religious beliefs (*N* = 53; 26%), cultural/religious diversity (*N* = 38;19%), “spiritual but not religious” moments (*N* = 84; 41%), dilemmas (*N* = 41; 20%) and doctors’ life experiences (*N* = 42; 21%). While most concerned patients, many were about physicians themselves (Table 2).

**Table 2:** Story Characteristics

**General Information**							**Totals**^**a**^
**Study Year**	1	1	1	3	11	20	
**Interview Batch**	**Batch 1** Class of 2003 – PGYs in 2001 (*n* = 13)	**Batch 2** Class of 2002 – PGY2s in 2001 (*n* = 10)	**Batch 3** Class of 2001 – PGY3s in 2001 (*n* = 11)	**Batch 4** Class of 2003 – PGY3s in 2003 (*n* = 13)	**Batch 5**^b^ Class of 2003 in 2011 (*n* = 12)	**Batch 6**^b^ Class of 2003 in 2021 (*n* = 7)	**All Interviews** (*n* = 66)
**Number of Stories**	31	26	27	57	42	21	**204**
**Average Story Per Interview**	2.38	2.6	2.45	4.38	3.5	3	**3.09**
**What are the stories about? (Most common situations/topics**							
**End of Life** *Fear of Death; Family Needs; Peace at Death;* *Futile ICU Care; Death of Child; Poor Prognosis; Rituals; Other*	8 (26%)	9 (35%)	15 (56%)	17 (30%)	8 (19%)	7 (33%)	**64 (31%)**
**Cultural/ Religious Diversity** *Patients with Unfamiliar Belief Systems; Language Barriers; Doctor with Strong Beliefs; Other*	9 (29%)	3 (12%)	8 (30%)	5 (9%)	11 (26%)	2 (9%)	**38 (19%)**
**Reproductive Health** *Abortion; Pregnancy; Birth Control; Other*	3 (10%)	2 (8%)	5 (19%)	4 (7%)	1 (2%)	1 (5%)	**16 (8%)**
**Other Clinical Situations** *Chronic Disease (eg. diabetes, COPD, chronic pain, obesity, chest pain, cancer); Mental Health (eg. depression, substance use disorder, grief, chronic anxiety)*	7 (23%)	1 (4%)	3 (11%)	4 (7%)	11 (26%)	1 (5%)	**27 (13%)**
**Patient’s Religion**	14 (45%)	7 (27%)	9 (33%)	10 (18%)	11 (26%)	2 (9%)	**53 (26%)**
**“Spiritual, Not-Religious” Moments** *Connection; Forgiveness; Coming to Peace; Personal Growth; Situations that feel spiritual but don’t know why; Music; Other*	6 (19%)	9 (35%)	11 (41%)	22 (39%)	19 (45%)	17 (81%)	**84 (41%)**
**Dilemmas—Boundaries, Role, Ethical**^c^ *Treatment Choices (eg. futile care); Regrets; Patient Asking Doctor about Their Beliefs; Requests for Prayer; Role of Doctor; Belief in Miracles; Other*	9 (29%)	6 (23%)	9 (33%)	7 (12%)	9 (21%)***	1 (5%)***	**41 (20%)**
**Doctor’s Spiritual Self-Care/Practices**	2 (6%)	2 (8%)	0 (0%)	5 (9%)	6 (14%)	4 (19%)	**19 (9%)**
**Doctor’s Life Experience Outside of Work** *Having Children; Death of Loved Ones; Marriage; Living in Other Countries; Leadership Experiences; Other*	5 (16%)	5 (19%)	6 (22%)	7 (12%)	7 (17%)	12 (57%)	**42 (21%)**
**Who are the stories about? (Characters)**							
**Patient**	16 (52%)	16 (62%)	15 (56%)	13 (23%)	23 (55%)	7 (33%)	**90 (44%)**
**Patient’s Family**	1 (3%)	0 (0%)	4 (15%)	4 (7%)	5 (12%)	2 (9%)	**16 (8%)**
**Physician**	6 (19%)	6 (23%)	10 (37%)	22 (39%)	14 (33%)	9 (43%)	**67 (33%)**
**Physicians’ Family**	4 (13%)	0 (0%)	2 (7%)	2 (3%)	1 (2%)	5 (24%)	**14 (7%)**
**Work Setting/Organization**	3 (10%)	3 (12%)	2 (7%)	5 (9%)	4 (9%)	4 (19%)	**21 (10%)**

^a^Each story often includes multiple topic categories and characters; therefore totals exceed 100%

^b^In Project Years 11 and 20, participants told individual stories as well as stories grouping many patients/situations together, reflecting significantly more patient care experience

^c^In Project Year 1 & 3, dilemma stories were often distressing; by Project Year 10, stories were more about challenges with strategies; and by Project Year 20 there were very few dilemma stories

### Thematic analysis

Three major themes emerged in physicians’ stories, and each evolved over time. Tables 3 and 4 provide examples of stories.

**Table 3:** Themes, subthemes and sample quotations

Theme	Subtheme	Representative Quotations
**Dissonance**	Internal Within doctor Within patient Within family member	“I don’t really understand it, but I found myself asking this patient whether she was having a suestal, and she said to me, ‘Yes.’ And I said, ‘Well, do you know anybody who could help you with this suestal?’ And she said, ‘Oh, I’m very religious. I’m not allowed to talk to people like that.’ So that gave me just a little bit of a glimpse into that [world]. She perceived herself as having experienced one of these events and yet the antidote to it, which is sometimes available, was not available to her because of her formal religious beliefs …”
		“I’m Catholic and I think that it’s meant different things to me at different times in my life … but I’ve come to really realize that certainly much greater than half of my patients in Central Falls or closer to 75% are at least baptized Catholic … I think that the way that we discuss certain topics regarding reproduction, regarding control of fertility … it’s almost as though there were absolutely no questions regarding what we are instructing patients to do. Like we don’t often ask patients who have just labored ‘Are you interested in using contraception?’ That’s not the question. The question is ‘What form of contraception are we going to tie you to before you leave this place?’ I really think it’s that strong. I’ve just recently come to think about these things and sort of question what my role in this whole thing is.”
	Inter-relational	“I felt that I was able to be a good support to her, to help her really grasp the fact that her father was being given a terminal diagnosis with a very poor prognosis … and she said ‘you’re Catholic, right?’ Because we did have a nice connection … and she wanted me to tell her that I was. And it was very uncomfortable in that moment; not only am I not Catholic … but I didn’t want to talk about what I was and what I wasn’t. It wasn’t about me …But she was looking to me for reassurance … but this was an area that I didn’t want to give it to her, didn’t want her to need that from me. I didn’t want to pretend to be Catholic just to gloss it over, but I didn’t want to begin to talk about my personal religious beliefs and I was really caught up as to how to continue that conversation with her … what did I do? I lied. I was caught off guard … It was the path of least resistance. And it didn’t feel good … I felt like I was falsely reassuring her so that she could talk. It was a falsehood – a white lie, if you will, but not one that felt right, but I didn’t know what my alternatives would have been in that interpersonal moment.”
	Systemic	“[She was] in her 20 s, … an IV drug user… [with] fulminant hepatic failure … It was clear that the family of this patient thought that she would somehow want to contribute something in her death, and they just felt this was such a tragedy that they wanted something positive to come out of it. And they said that she particularly wanted to donate her body to science… I brought that up with the medical team, and they completely shot down the idea. They had a very narrow view of what they meant by donating her body to science, and the family’s view was that they wanted her body to be donated someplace to do research to find out how to get a cure for this, and they said, “No one is going to want that body,” and that’s essentially what they told the patient’s family.”
**Formation of the Physician**	A physicians’ work is inherently spiritually weighty	“My christening moment as a doctor was as a third year medical student when I had a patient with diabetes whose kidneys were acutely failing and who had a fulminant infection that the only treatment would further compromise his kidneys and I took care of him for several weeks, but one particular morning I went in and he seemed just really frightened and we spent probably half an hour talking about his fears of death and what that meant to him and his family … and later that day we all walked in with all the team … the nephrologist, the internist, two residents and me the medical student … and they were having a very serious discussion with him about what his prognosis was and what his options were and at the end of all of it he looked straight at me and said ‘What do you think I should do? I trust you’ I recognized that of all those people, I was the person who had invested the time to talk to him about what was really important to him at that point in his life. He went into a coma a couple of days later and his family was at his bedside and for the first few days I couldn’t enter the room. I would walk by it and feel that draw, that kind of gravitational draw that I needed to go in and talk to his family and I didn’t know what I was going to say to them. Finally, I went in one day when his daughter was there, and he had talked to me a lot about his daughter, and I looked at her and she looked at me and we both just started crying. We spent about ½ hour together at his bedside and I conveyed to her some other conversations that I had with him and what his ideas were about life and it was just a wonderful moment for all of us … **[These moments] are in the end what rejuvenates you … I am very aware as a physician that you have access to incredible moments in people’s lives and very spiritual moments and what an incredible privilege that is, but also how much responsibility comes with that.”**
	Physician as a “Work in Progress” with their own professional and spiritual journey	“He seemed very at peace with using Hospice to pass on, at home, very dignified, with his family…It was still against my knee-jerk reaction to think, ‘We can do more, we can do more.’ But we were doing something, it just wasn’t the something that I perceived as being the right thing for him. I guess that spirituality helped him make the decision that he really wanted.”
	The value of reflection	“Having had a baby recently, for example, has changed so much. I had had no awareness of what it was like, even though I had been seeing prenatal patients and delivering their babies, and even seeing the babies in clinic afterwards. Really grasping what it’s all about is at a whole different level once you go through it… That’s why I bring up having a baby because you don’t always know what it’s like until it happens to you. But there are all these parallels that you can draw upon to try to better understand it.”
**Healing**	Healing for patient	“She came into the office with chest pain. She had called earlier in the day, and refused to go to the Emergency Room. So, I went in, mad at her. If she really had chest pain, I was wasting my time and I was wasting her time. I started to take the history. The truth ended up being that she was really depressed about her brother dying. It was the one-year anniversary of his death… I said, ‘Do you want me to give you a medicine for this pain?’… She was like, ‘Oh, no. It’s gone.’ So, there you go. I came out of the room feeling rather triumphant and then I said, ‘Well, I just cured your chest pain.’ So obviously, sometimes that’s not the reason, but if you’re not compassionate, then you’re never going to get that story. Then, you’re going to send that poor woman who I was irritated at to the ER to get a full $1,000 work-up of chest pain, that was her brother dying, and she needed to talk to someone. I am always impressed by that. In most people, just sort of getting it out there allows healing.”
	Healing for doctor	“[I am not religious, but] … I don’t know if I should say this … [there was this time] I really experienced God personally in my life. God was in the room. I really felt that … [The University] had brought in this speaker … she was an African American woman who had some terminal disease … and she gave a speech, and as she was doing that she actually made up a song, … and she actually had everyone in the audience singing with her … [later] she started singing again and had us singing with her. And that was even more intense, because just like everyone knew the words. And I was really overcome, and I started to cry… That was extremely spiritual… It was unbelievable that she had this terminal disease and there she was leading us, uplifting us. It was like walking away from time”
	Healing between doctor and patient	“[My patient] lost a baby at about 30 weeks, and she was devastated … And her family and their faith, was even beyond the concept of religion. It was totally sustaining for them. It was the first thing we talked about in the first minutes I saw them. And that was very satisfying, to connect with someone on a human level in a way that was very important to them. And it was good for me.”
		“When you are in training, you question your own abilities … hindsight is 20/20 … a young woman in her 40’s … single mother, divorced, mother of two teenagers … She had a history of colon cancer … treated … and cancer free … I went away on vacation … they found on CT Scan a large tumor … I thought … ‘God, if I had only done something sooner. If I had just done the CT scan 2 weeks ago’ … She never was pointing fingers. She never blamed me for not catching it earlier … I took care of her in the hospital when she was really sick … I had long conversations with her in the hospital. Just her dealing with her own mortality. She was so strong … her thoughts were more on her family. She had strong religious beliefs, and we talked about that a lot … She ended up going to hospice and dying within a couple of months … I hope my presence there was healing for her. I know that it was healing for me because I was dealing with my own insecurities.”
	Collective healing experiences	“One of the first times where I saw a rapid deterioration in one of my [continuity] patients … in his spare time he was an organ player in his church … he was a religious person and loved music as well … he ended up dying in the ICU … but he was still ‘with it’ enough towards the end, the church choir came to his bedside and sang for him … It was an unexpected death for him … but his wife was there and the people he was closest to were there, and the music was there, his religion was there, and I thought that was great.”

**Table 4:** Thematic progression over time

Thematic Progression	Representative Quotations
Theme 1: Dissonance to Integration	“I do a lot of refugee health, this is a large refugee family who came from the Congo … and I take care of the whole family, parents and kids … the mom is in her mid 40 s and her youngest is like 2 or 3 years old … She came in with abdominal pain and weight loss … ended up being diagnosed with metastatic cancer …. [with only] months left. And she was at the cancer hospital for a week and then sent home with home hospice services. And the family came in to see me and said you know we don’t know what’s going on, you’ve know us for so long, there must be something you can do … we can’t just take her home to die. I mean it was just one of those really, you know on a short list of sort of heartbreaking times for me as a physician because it felt like there was so little in terms of, you know nothing I could add in terms of factual information about her prognosis which is incredibly poor. And you know, again it was just about kind of being comfortable sitting in that space with them and crying with them, which I did—something I definitely do with patients, I mean not on a daily basis or anything, but something I do … I’ve prayed with patients, like when patients ask if I’ll pray with them which you know usually just being sort of closing my eyes and holding hands and that kind of thing. And just sitting there and bonding in that state…”. (Y20/P12)
Theme 2: Formation and Transformation	**Formation as Ongoing:** “I think each of the experiences that you have in life is in a certain direction, and all of those experiences impact how you look at things … I had sort of Christian influences growing up … and I had some Buddhist influences of watching my uncle through the process he went through [substance use disorder], and I ended up with a pretty sort of universal thinking about spirituality. Spirituality is a human thing, it’s a need that, you know, everybody manifests a little differently but it all comes back to sort of the basic principles and if you concentrate on the similarities and not the differences.” (Y20/P3)
	**Relational Transformation** “My population is aging with me. So, a good portion of my patients, either they or their family members have been a part of my life for more than 10 years and have followed me to other locations … for them my comfort in discussing their faith is, it’s easy, it’s very natural … with newer patients you test that relationship, and that comfort level … that kind of depth can develop with time. Although I did have a brand new patient yesterday and we talked about spirituality, why he thought he survived so many traumas in his life, and to be 35 and how does he feel God sees him … and we had that conversation … and he was someone I had never talked to before … So, I’m probably more confident than I was, definitely with my old timers but even I guess with my newer patients. Because I know how successful it has been, and how rewarding it has been to have those conversations time and again … so it’s not just something that I was told is good and should be done because it’s good, but that I’ve experienced that, and that makes it easier. Yeah, seeing the fruits of that over the years. (Y20/P8)
	**Personal Transformation** “I think all of our life events have influenced us but probably the most defining one, which I presume I mentioned in previous interviews, was my mother’s very sudden death when I was a medical student. And that was between my third and fourth year of medical school. My mother fell suddenly in July … She went from a pretty healthy functioning 57 year old to dead in the space of about 10 days from unknown, to a bleeding ulcer in her stomach, to you know, full arrest. So having that experience, and it was a month before my wedding, having that experience at that time in my life stripped away a lot of the bullshit and allowed me to be more present to ridiculous suffering. Yeah, yeah, just unspeakable loss. So I think that experience early in my medical training and reasonably early in my life was defining in my capacity to develop my own spiritual core. And to be present with other people in suffering … And in the middle of that, I actually converted from Catholicism to Judaism [prior to marriage]…so I have this vivid memory of almost 3 weeks after my mother’s death, a week before my wedding, going to a ritual bath … and the friend that came with me was reading from Genesis, as I submerged myself in the mikveh, and there were seven steps to get down to the pool. So she was reading from her little Palm Pilot Genesis, the seven days of creation and with each day of creation I took a step further into the bath. And that whole period of time, that whole crazy haze was transformative and anchoring, and certainly I have grown since then and certainly my spiritual life has evolved, I guess. But I very much still identify with that woman at that point in time.” (Y20/P5)
	**Systemic Transformation** “Before we went into the rooms they would stop and say, all right, before we go in, take a deep breath, we're going to send this patient light and love. You all take a deep breath, close your eyes, send positive energy, and then go into the room … that’s just, I think, a different experience than what many experienced on rounds in the hospital. Then if we weren’t doing walk rounds we were doing sit down rounds, we started with a moment of silence, just like two to five minutes to deep breathe or meditate or reflect on a poem or something just to sort of acknowledge we’re human. This is hard. We’re all in this together … Those have been real gifts … Now I work for an insurance company, and the culture is very different and I try to bring a little bit of that you know. We have weekly team rounds where we talk about patients and I’ve started those by putting up a quote on the Zoom before the rounds to try to acknowledge the work that people are doing … I’m not quite comfortable enough to have them do meditation or breathing over zoom, it’s hard. You don’t know what people are doing …email.. rolling their eyes… But that’s been one of the things that I’ve been trying to tip toe into and what I find is that people so much, just want connection and so much want to be acknowledged. And you know, typically, if you don’t get in too much to one religion or the other, people really want that type of connection; they want to be human” (Y20/P3)
Theme 3: “Accidental” Healing to “Purposeful” Healing	“It was a real bonding experience … and we ended up talking for an hour and a half; because how do you leave the room with someone pregnant telling you they’re afraid they are going to die and that there is voodoo in the air? … in her culture, people really felt that people can really wish evil …[she had] premonitions … she was going to have a postpartum hemorrhage and die … [I asked] what kind of support do you have? Because her husband who was like a priest did not believe in voodoo. And she identified a priest in the community who understood these forces, and they had a prayer ceremony … [afterwards] she came back and was relaxed and comfortable to deliver the baby.”(Y1/PGY3/PF)
	“Working in a prison … I chose it intentionally to deepen my practice of compassion … I hold the conviction that everybody is worthy of compassion, love and relief of suffering, then if I thought they were worthy of it, why wasn’t I giving it? So, there are certainly times when I feel like I was the only person on the planet offering compassion to the individual I was sitting with. And I think that is healing … So, I actually carried … this classic book “Be Here Now” … it’s very sort of 70’s style but it is a classic … and I would keep it under my driver’s side seat … So, I would drive to the prison, I would park in the parking lot and I would … read some before I would go into clinic. And every time—there are a lot of locked doors you have to go through as you're heading into my office, which is deep in the prison. And every door you have to go through you have to stop … and wait while they buzz you in … And I used that as my meditation trick – each door I would do a breathing meditation (laughs) before I walked through. It was really, it really helped me get in the right frame of mind for the time when I finally got to my clinic.” (Y10/P10)

### Theme-1: Dissonance to integration

Many stories involved experiencing or witnessing dissonances – conflicts between beliefs and experience – at internal, inter-relational, and/or systemic levels. Dissonance occurred due to discordance between physician or patient beliefs and the care they gave or received. At times, additional systemic structural factors prevented physicians from acting in accordance with their values. Stories of dissonance were more commonly told by physicians during residency training and dissipated over time as physicians transitioned to leadership roles and integrated their values into their practice.

#### Subtheme-1: Internal dissonances

Participants often described conflicts within themselves, patients or family members. One shared a patient’s struggle with meaning: *“… [She] had intrauterine fetal demise. And she was very distressed about it … she was clearly a religious person, asking herself, “Why? Why did God do this?”* Conflicts within doctors were wide-ranging, including disconnects between personal beliefs and routine care or questions regarding boundaries of role. One participant shared: *“…every time I talk to her she says, ‘pray for me’ … and [I wonder] ‘Should I? How do I do that? … I try to incorporate [spiritual care] as much as I can when they want that. But can you not be helpful to a patient spiritually if you yourself are not spiritual?”.*

#### Subtheme-2: Inter-relational dissonance

Conflicts within doctor-patient, patient-family, and doctor-family relationships frequently involved treatment choices, especially towards end-of-life: *“… a gentleman from Ghana … had really bad laryngeal cancer, and he needed surgery … there was something about the act of cutting his body that was disturbing to him from a spiritual point of view … yet from a medical point of view … it seemed that was really the best option for him… He decided not to have the surgery … I think that was really the hardest for me – accepting that decision, particularly when he came back [later] with advanced cancer…”* Others shared stories of belief in miracles clashing with medical recommendations and of the discomfort associated with being asked about their own beliefs by patients/families.

#### Subtheme-3: Systemic dissonances

Physicians frequently described what they identified as spiritual conflicts with the medical system (hierarchies, time-crunches, values). One participant, a resident, described a harrowing incident of moral distress when respecting hierarchy clashed with their values regarding honesty and autonomy: *“I had to intubate a patient who had said [many times] she didn’t want to be intubated [again] and the attending physician came in and said ‘I promise you I’ll take this out in three days if this doesn’t work’ … she didn’t respond and he walked out of the room and said ‘put the tube in.’ And when three days came, he didn’t pull it out … I kind of wish I had just said, ‘You know what? I’m not intubating her’ … But the thing was, he lied to her.”* This type of deep ethical dilemma was more frequent in residents’ stories than in practicing physicians’ stories, reflecting residents’ perceived lack of power within the medical hierarchy coupled with their front-line responsibility to write orders and/or perform procedures.

#### Subtheme-4: Changes over time—dissonance to integration

As participants gained experience, accounts of dissonances notably reduced as many found patient-centered ways of integrating spiritual care into their role as physicians. Several noted positive effects for patients: *“My sense is that it brought her worlds together… when she was going through chemo … there was that world, you know, the medical world and then there was the home community, which was her spiritual world, and I felt like my office was a place where both of those could coexist.”* As participants progressed into leadership roles, several worked to reduce systemic dissonances. (See additional and expanded stories in Tables 3 and 4).

### Theme-2: Formation and transformation

Many participants spoke of how events they identified as spiritually impactful had been critical to their growth as physicians and humans. These formative experiences ranged from personal to professional, spiritually distressing to spiritually healing, and occurred during all study-years. As physicians progressed in their career, many underwent “transformation” – their stories demonstrated a greater capacity to understand and care for the spiritual needs of their patients, their team, and themselves.

#### Subtheme-1: The spiritually weighty work of physicians

Many participants described their work as inherently spiritually weighty. As residents, participants frequently told stories of spiritual distress and responsibility when caring for dying patients: “… ‘*Doctor, do you think I’m going to die?’ I think that was the first time that anyone really had asked me that. Your first response is to try to comfort them…’No, I don’t think you’re going to die anytime soon.’ … Well, he died the next day…I felt that maybe I hadn’t been honest with him…Maybe he knew something I didn’t know … Maybe he would have wanted to spend his last hours in a different way, had I told him something else … It was indelibly etched in my mind.”* Later in practice, when much of the uncertainty of inexperience had faded, physicians continued to tell stories of spiritually weighty experiences. During the COVID-19 pandemic, some spoke of the spiritual toll of losing patients, colleagues, and friends.

#### Subtheme-2: Value of reflection and self-care

The importance of time to reflect upon deeply impactful experiences and spiritual self-care—ranging from religious practices to time with family—was expressed across all study years, especially study-years 11 and 20. Physicians’ stories illustrated how crucial spiritual self-care was for maintaining compassion and addressing patients’ diverse needs. *“I've become a big student of yoga … [This has] really helped me a tremendous amount in understanding my own needs and being able to restore my capacity for empathy … figuring out how to take care of people but also take care of myself. That was a journey for me that didn't come naturally.”*

#### Subtheme-3—Physicians as works in progress

Several participants discussed personally formative moments, often after residency, that impacted their spiritual growth and clinical approach: *“I have kids, and that showed me a lot about how little control you have over anything. … I have lost dear loved ones … it's really different on the other side of that divide. So, I do think that has informed a lot of how I approach things … just actually listening to patients … that has also led me to a lot of different ways to try and heal people.”*

Some participants told the same impactful story on subsequent interviews, years later, each time with slightly different interpretations. For example, one participant shared: *“…my mother’s sudden death when I was a medical student … it was a month before my wedding. Having that experience at that time in my life stripped away a lot of the bullshit and allowed me to be more present to ridiculous suffering … [it] was defining in my capacity to develop my own spiritual core. And to be present to other people in suffering.”*

#### Subtheme-4: Change over time—transformation

As participants progressed in their careers, many experienced three types of transformation related to spirituality and medicine: relational, personal, and systemic. Relational transformation was commonly the first to emerge, with participants demonstrating greater capacity for understanding patients’ diverse spiritual needs. While personal transformation varied, several participants described an integration of their clinical practice into their meaning-in-life/spiritual practice: *“I believe that my choice to be a physician is a spiritual calling, and so I think just renewing that choice, reflecting on how similar I am as both a mother, and as a woman, as a patient, as a citizen of New York, that all of these contribute to … the need for me to be here now [New York City; pandemic] doing the things I am doing, that there is a reason for that.”*A few participants engaged in systems transformation to integrate spiritual health into their medical organizations. (See Tables 3 and 4).

### Theme-3: Healing

Healing—at individual, inter-relational, and collective levels—was a common theme in participants’ stories. While some participants spoke of religious sources of healing, many identified non-religious forms of spiritual relief and reconstitution. Over time, healing practices became more intentionally incorporated into a physician’s practice.

#### Subtheme-1: Individual healing

Several participants described how patients derive healing from spiritual beliefs: *“I was struck by their intense faith, it was just something where I can remember being sort of jealous of them, because I didn’t have that … then unexpectedly the mom died … it was unbelievable what they had gone through, the loss …yet they still had this really firm faith.”*

Others shared how they and their work benefited from consistent spiritual self-care, and still others described profound healing moments: *“ … she started singing again and had us singing with her. … I was really overcome, and I started to cry… That was extremely spiritual… she had this terminal disease and there she was leading us, uplifting us. It was like walking away from time”.*

#### Subtheme-2: Inter-relational healing

Many participants remarked that communication, trust, and connection were important tools for healing. Several physicians’ stories demonstrated how they themselves, not just patients, felt positive effects from these healing encounters: *“When you are in training, you question your own abilities … I went away on vacation … they found on CT scan a large tumor … I thought … ‘God, if I had only done something sooner’ … She ended up going to hospice and dying within a couple of months … I hope my presence there was healing for her. I know that it was healing for me because I was dealing with my own insecurities.”*

Participants identified that recognizing one's role as a healer, and not a mere technician, led to contentment, rejuvenation and sense of meaning: “*There was this moment … where she sort of took my hand and thanked me for everything. It was a moving experience. It was not so much a religious experience, but it felt like I had gotten through to her. It was the first time I felt like I had been important to her …”.*

Others described the power of inter-relational grieving: *“My mother was sick…We were on the verge of withdrawing care … The surgeon who had taken care of her … came in to see her while I was there. He was quiet… I saw tears in his eyes… and he said ‘You know, my mother just passed away’ … We were both there for a while, quietly… That was a very healing moment for me because it made it very human in the sense of the medical people and the patient people being the same people.”*

#### Subtheme-3: Collective healing

Several participants told stories of collective healing experiences. Music/chanting was frequently mentioned: *“They brought in a whole community– his family and the spiritual [Buddhist] community. They chanted for about an hour in the ICU… so this chanting was just floating around in the ICU. Nobody did anything. There was work to be done. Nobody was horribly ill, but there was paperwork… [We all] just sat there and listened to the chanting…”* Other stories illustrated how the use of brief spiritual/grounding techniques gave medical teams a sense of healing and community, decreasing participants’ loneliness.

#### Subtheme-4: Changes over time—accidental to purposeful healing

Over time, most participants developed ways to consciously integrate spiritual health into medical practice. Many described finding community spiritual care resources for their diverse patient populations. Some utilized their own spiritual practices to enhance compassion: *“There are patients that are very challenging for me … Before I go into the room, I fill myself with a deep breath and a mindset, ‘Ok, my work is important’ and I find a place of compassion, or a sense of generosity towards someone who may be ‘difficult’ – not towards me, but because their life is difficult. So that, on an internal level, is the way I find that being spiritual in my approach to medicine is helpful.”*

Some, in leadership roles, found inclusive, secular methods for incorporating healing and spiritual well-being into their practice environment. *“I encouraged our organization to create a policy … [for] when a patient dies … now [we have] added a component which I believe is spiritual for the team, and care of ourselves … we take a moment and acknowledge a gratitude for them [patients] in our life, and for the opportunity to serve them … it's very non-denominational language … I felt very happy to feel comfortable bringing that to this organization … to recognize this spiritual part of the work that we do.”* (See Tables 3 and 4).

### Relationship between themes – theoretical model

**Fig. 1:**
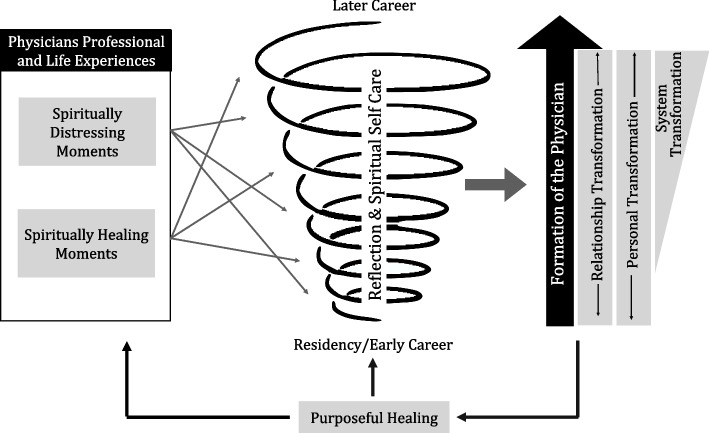
Relationship between themes

Axial and selective coding analysis resulted in a theoretical model of relationship between themes and changes over time (Fig. 1). The first part of the figure illustrates that both spiritually distressing and spiritually healing moments occur throughout a physician's professional and personal life and are integral to their identity formation over time. The center part of the figure illustrates how reflection on these moments combined with spiritual self-care can foster the progression of physician formation towards a deepening of self-awareness, resolution of conflicts related to values and beliefs, resilience, and sense of meaning and agency. This progressive physician formation appears associated with transformation at the personal, relationship, and systems levels.

In our study, relationship transformation occurred most frequently, linked with physicians’ desire to better understand the comprehensive healthcare needs of their patients. Many physicians’ stories also reflected personal transformation of various sorts and some physicians’ stories reflected their efforts towards systems level transformation, particularly later in their career. Finally, as physicians progressed in their personal/professional formation, many appear to transition from being “accidental healers” to “purposeful healers”, positively impacting their own response to spiritually distressing and spiritually healing moments, the way they responded to patients’ spiritual needs, and the way they interacted with organizational culture.

## Discussion

This qualitative analysis of physicians’ stories about spirituality and medical practice over 20-years reveals new insights into participants’ experiences of meaning, purpose, personal growth, relationships and healing as they progress through their career. By allowing participants to define spirituality for themselves and having a high response rate, we captured a broad range of experiences, from secular to religious. To our knowledge, no other study has explored physicians’ longitudinal experiences in this way. Our study adds new insights into three specific fields – (1) narrative medicine, (2) adult learning theory, particularly transformative learning and professional/personal formation; and (3) physician burnout.

Narrative or “story” has long been recognized as a powerful way to tap into levels of human experiences that are not readily apparent in everyday life [68–74]. In narrative medicine, storytelling is a means for understanding the emotional challenges physicians face and is utilized for expanding physician empathy and humanism [70–73, 82]. However, studies in this field rarely explicitly discuss spiritual themes.

In “The Power of Myth,” Joseph Campbell [68] explores how storytelling is used by spiritual traditions worldwide to understand issues of ultimate meaning, deep beliefs, purpose and values – issues that become paramount when confronting suffering and death. By explicitly examining physicians’ stories regarding what they define for themselves as spiritual, we gained a unique window into physicians’ experiences beyond the cognitive and emotional levels to the more elusive realm of spiritual health and well-being.

Our study also provides new insight into transformative learning and formation in physicians. Transformative learning, “a learning process of making meaning of one’s experience,” [83] is often discussed in undergraduate and graduate medical education, along with curricular innovations to build learner resilience, well-being and compassion [83–85]. The spiritually impactful moments study participants shared, related to their work witnessing human suffering and death, within imperfect systems, are critical experiences that can trigger transformative learning [83, 86].

Many authors discuss countering negative effects of the “hidden curriculum” that arise when “real life” clinical and systems experiences conflict with ideals of humanism espoused in the classroom, resulting in an erosion of values and ideals [87, 88]. However, Martimianakis et al. [87] argue that this approach “obfuscates the positive effects of … informal learning, including the many ways that educators and learners learn to navigate contradictory messages about what is right and what is possible, while managing, and perhaps most important, challenging the realities of contemporary health care.”

Our study may have inadvertently served as an intervention in transformative learning, supporting Martimianakis’s argument. It is possible that being interviewed longitudinally may have triggered participant reflection on spiritually complex experiences, fostered resolution of conflicts between values and systemic barriers to patient-centered care, and for some, inspired leadership in systems change. This hypothesis requires testing in future studies.

Transformative learning is central to how work and life experiences influence physicians’ personal and professional formation. While previous studies have identified emotionally fraught moments as formative for physicians [10–18, 75, 76, 89–97], few, if any, have examined the power of spiritually healing moments. Merriam, Tisdell and others [86, 98] argue that attending to spirituality in adult learning is important for “authenticity in identity development.” By asking participants directly about spirituality, as they defined it, our study revealed a wide range of formative moments, both healing and distressing. The notion that examining spiritually healing moments may help balance effects of distressing situations, is supported by the work of Pargament, Pomerleau and others [27–29], and warrants further study.

While previous studies define physician formation as an iterative, lifelong process, most only examine formation in medical students and residents [73, 89–93, 95–97]. Our study’s unique longitudinal nature provides insight into formative experiences occurring later in life, impacting participants’ roles as physicians, leaders, mentors and teachers. Some stories told and re-told across study years, illustrate how meaning-making and healing of wounds that physicians carry can sometimes take years to fully process and resolve.

As study physicians progressed in their careers, stories revealed transformation at three levels: personal, inter-relational, and systemic. These are consistent with physician formation studies rooted in developmental psychology [89] and mirror the four central physician narratives: physician–patient, physician-self, physician-colleague, physician-society [70, 77]. Systems transformation appeared in later stories as some physicians, now in positions of authority, intentionally fostered healing work environments.

Our study provides new insights regarding physician burnout [1–19, 75, 76]. Two of three domains of Maslach’s Burnout Inventory [19] are consistent with spiritual distress – (a) lack of self-efficacy, related to inability to find meaning in one’s work and (b) depersonalization, reflecting lack of connection. Our finding that reflecting on both spiritually healing and distressing experiences can enhance meaning-making and connection, is supported by recent studies on meaning-making as an effective strategy for burnout prevention in healthcare providers [20, 21], and studies on burnout and spirituality in learners [99–101].

These findings are also consistent with work in the field of psychology related to spiritual struggle [27–29]. Numerous studies demonstrate that stressful events trigger spiritual struggles when an individual’s most deeply held beliefs and values are shaken. Several explore the mechanisms by which such things as meaning-making, compassion and quest can transform spiritual struggle into psychological growth, potentially preventing progression to chronic anxiety, depression or post-traumatic stress disorder [27–29]. A similar mechanism might be relevant for burnout prevention in healthcare providers and warrants further study. Our study provides new data supporting the theory that broadening the focus from meaning-making alone (cognitive/existential domain) to healing moments that tap into a deeper spiritual level, like forgiveness, hope and connection (experiential domain), has the potential to foster physician spiritual well-being and resilience in multiple dimensions [22, 41, 102, 103].

### Limitations

Study participants all attended the same family medicine residency. While over 20-years they had practiced in many settings, findings may not be generalizable to other physicians. Additionally, trajectories of physicians who received basic spiritual care training during residency may not be reflective of physicians without training. However, all 34 participants in study-year 1 (no spiritual care training) had rich stories, indicating the common occurrence of spiritually impactful clinical experiences.

## Conclusion

This study, unique in the medical literature, reveals that physicians, both secular and religious, have rich stories to tell regarding spiritually distressing and healing moments throughout their careers. Active reflection on these experiences, along with spiritual self-care, may positively impact physicians’ personal and professional identity formation by fostering meaning-making and deeper connections with self and others. This could enhance job satisfaction and mitigate burnout in both practicing physicians and trainees. Medical educators can play an active role in this process by expanding narrative medicine, professional identity formation, and wellness initiatives throughout the educational spectrum to include consideration of the spiritual dimension.

## Data Availability

The data that support the findings of this individual interview study are not openly available due to reasons of participant anonymity and topic sensitivity, as delineated in the IRB requirements and approval process for this study. Data can be available on reasonable request only if the specific request is approved by our Institutional Review Board. Note: This is not a clinical trial, so no clinical trial number is available.

## Abbreviations

PGY: Post Graduate Year (Residency Year)
RR: Response Rate
IRB: Institutional Review Board
ICU: Intensive Care Unit
